# 1,3,5-Tris(*N*-phenyl­benzimidazol-2-yl)benzene methanol solvate

**DOI:** 10.1107/S1600536809036046

**Published:** 2009-09-12

**Authors:** Wei-Feng Song, Ying Wu, Yan Fan, Yue Wang, Yu Liu

**Affiliations:** a State Key Laboratory of Supramolecular Structure and Materials, College of Chemistry, Jilin University, Changchun 130012, People’s Republic of China

## Abstract

The main mol­ecule of the title compound, C_45_H_30_N_6_·CH_3_OH, has a non-planar core: the dihedral angles between the benzimidazole rings and the central benzene ring are 20.19 (10), 34.57 (8), and 44.59 (8)°, while the dihedral angles between the peripheral phenyl rings and the attached benzimidazole rings are 84.57 (7), 62.71 (6) and 51.73 (6)°. The tri-substituted benzene mol­ecule is connected to the methanol solvent mol­ecule through an O—H⋯N hydrogen bond, forming a 1:1 solvate. In the crystal structure, no significant π–π inter­actions are present, and the mol­ecules are associated through weak C—H⋯N and C—H⋯O(methanol) contacts.

## Related literature

For OLEDs (organic light emitting diodes), see: Adachi *et al.* (2001 ▶); Gao *et al.* (1999 ▶); Shi *et al.* (1997 ▶); Lo *et al.* (2002 ▶). For the structure of a related solvate, see: Totsatitpaisan *et al.* (2008 ▶). 
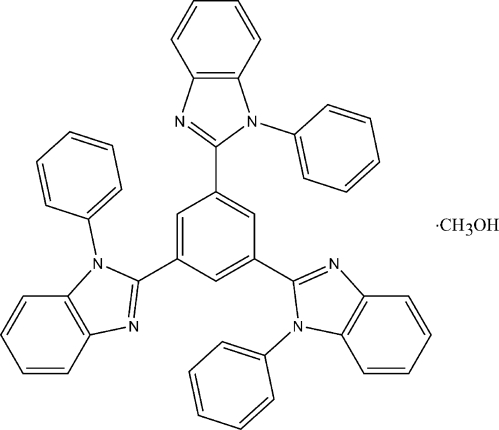


## Experimental

### 

#### Crystal data

C_45_H_30_N_6_·CH_4_O
*M* *_r_* = 686.79
Monoclinic, 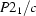

*a* = 11.253 (2) Å
*b* = 18.692 (4) Å
*c* = 17.763 (4) Å
β = 101.58 (3)°
*V* = 3660.1 (13) Å^3^
*Z* = 4
Mo *K*α radiation
μ = 0.08 mm^−1^
*T* = 293 K
0.26 × 0.20 × 0.19 mm

#### Data collection

Rigaku R-AXIS RAPID IP diffractometer
Absorption correction: multi-scan (*ABSCOR*; Higashi, 1995 ▶) *T*_min_ = 0.981, *T*_max_ = 0.986
35250 measured reflections
8285 independent reflections
4831 reflections with *I* > 2σ(*I*)
*R*_int_ = 0.058

#### Refinement

*R*[*F* ^2^ > 2σ(*F* ^2^)] = 0.051
*wR*(*F* ^2^) = 0.142
*S* = 1.01
8285 reflections
480 parameters
H-atom parameters constrained
Δρ_max_ = 0.14 e Å^−3^
Δρ_min_ = −0.19 e Å^−3^

### 

Data collection: *RAPID-AUTO* (Rigaku, 1998 ▶); cell refinement: *RAPID-AUTO*; data reduction: *RAPID-AUTO*; program(s) used to solve structure: *SHELXS97* (Sheldrick, 2008 ▶); program(s) used to refine structure: *SHELXL97* (Sheldrick, 2008 ▶); molecular graphics: *SHELXTL* (Sheldrick, 2008 ▶); software used to prepare material for publication: *SHELXL97*.

## Supplementary Material

Crystal structure: contains datablocks global, I. DOI: 10.1107/S1600536809036046/bh2239sup1.cif

Structure factors: contains datablocks I. DOI: 10.1107/S1600536809036046/bh2239Isup2.hkl

Additional supplementary materials:  crystallographic information; 3D view; checkCIF report

## Figures and Tables

**Table 1:** Hydrogen-bond geometry (Å, °)

*D*—H⋯*A*	*D*—H	H⋯*A*	*D*⋯*A*	*D*—H⋯*A*
O—H0⋯N1^i^	0.82	2.12	2.936 (2)	170
C25—H25⋯N5^ii^	0.93	2.69	3.603 (3)	169
C37—H37⋯N4^iii^	0.93	2.43	3.338 (3)	164
C32—H32⋯O^iv^	0.93	2.71	3.605 (3)	161

Symmetry codes: (i) 
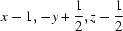
; (ii) 
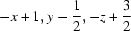
; (iii) 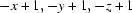; (iv) 
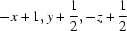
.

**Fig. 1.**
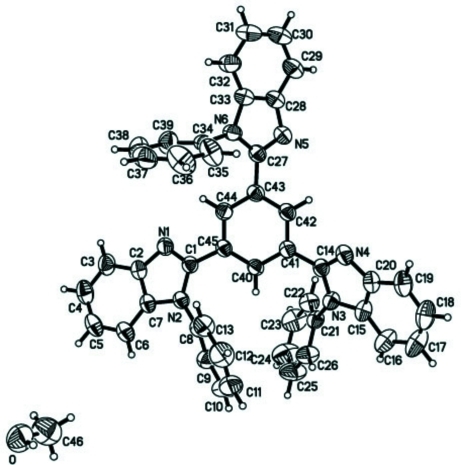
The molecular structure of the title compound. Displacement ellipsoids are drawn at the 50% probability level.

**Fig. 2.**
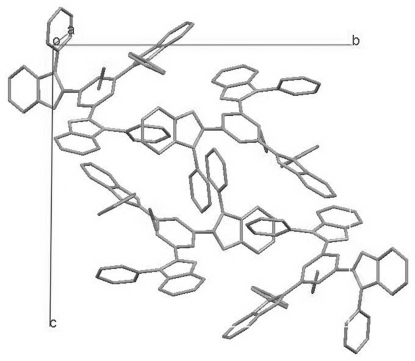
The packing of title compound along the a axis.

## supplementary crystallographic information

### Comment

For many years there has been extensive research on OLED because of their high luminance, low drive voltage, fast response time, wide viewing angle and a variety of emission color in flat-panel displays. To achieve highly efficient organic electroluminescence, it is essential to confine the excitons within the emitting layer and prevent the excitons and holes to approach the cathode. Therefore, an electron transporting/hole blocking layer is often introduced between the emitting layer and the cathode in some organic electroluminescent diodes (OLEDs). Due to its almost omnipotent properties, TPBI, 1,3,5-tris(2-*N*-phenylbenzimidazolyl)benzene, is an excellent electroluminescent (EL) material, which has been used as an electron-transporting layer for OLEDs based on fluorescent emitters (Gao *et al.*, 1999), as host (Adachi *et al.*, 2001) or electron-transporting/hole-blocking layer (Lo *et al.*, 2002) for phosphorescent emitters, and enhanced the efficiency of OLEDs. For these reasons, it has attracted considerable interest for years. However, its crystal structure has not been reported until now.

As shown in Fig. 1, the molecular skeleton is non-planar. The dihedral angles between the benzimidazole rings and the central benzene ring, and between the peripheral phenyl rings and the benzimidazole rings, are 20.19 (10), 34.57 (8), 44.59 (8)° and 84.57 (7), 62.71 (6), 51.73 (6)°, respectively. The bond lengths and angles are all in normal ranges. The TPBI molecule forms a 1:1 solvate with methanol, a situation reminiscent of that reported for an other TPBI derivative, stabilized with 2-propanol (Totsatitpaisan *et al.*, 2008).

The packing of the title compound (Fig. 2) shows that there is no π···π interactions. Instead, the molecules are connected with hydrogen bonds.

### Experimental

To a solution of *N*-phenyl-1,2-phenylenediamine (16.6 g, 0.09 mol) in 100 ml of *N*-methyl pyrrolidinone was added 1,3,5-benzenetricarbonyl chloride (8.0 g, 0.03 mol) in portion at room temperature under nitrogen. The reaction mixture was stirred at room temperature for 2 h, then raise temperature to 50 for another 0.5 h. After cooling, the mixture was poured into 300 ml of cool water with stirring. The resulted precipitates were filtered and washed with water. After drying, the tribenzamide was collected to give 19.5 g. The TPBI [1,3,5-tris(2-*N*-phenylbenzimidazolyl)benzene)] was obtained by heating the tribenzamide in 0.3 atm, nitrogen pressure, at 553–568 K for about one hour. The pure TPBI was obtained by sublimation twice at 588 K at 2 Torr pressure. Colorless crystals suitable for X-ray diffraction study were obtained by slow evaporation of a methanol solution.

### Refinement

H atoms were placed in calculated positions, with aryl C—H distances of 0.93 Å and methyl C—H distances of 0.96 Å and were refined using a riding model with *U*_iso_(H) = 1.2*U*_eq_(aryl C) or 1.5*U*_eq_(methyl C).

### Crystal data

**Table:**

C_45_H_30_N_6_·CH_4_O	*F*(000) = 1440
*M**_r_* = 686.79	*D*_x_ = 1.246 Mg m^−^^3^
Monoclinic, *P*2_1_/*c*	Mo *K*α radiation, λ = 0.71073 Å
Hall symbol: -P 2ybc	Cell parameters from 20230 reflections
*a* = 11.253 (2) Å	θ = 3.2–27.4°
*b* = 18.692 (4) Å	µ = 0.08 mm^−^^1^
*c* = 17.763 (4) Å	*T* = 293 K
β = 101.58 (3)°	Block, white
*V* = 3660.1 (13) Å^3^	0.26 × 0.20 × 0.19 mm
*Z* = 4	

### Data collection

**Table:**

Rigaku R-AXIS RAPID IP diffractometer	8285 independent reflections
Radiation source: fine-focus sealed tube	4831 reflections with *I* > 2σ(*I*)
graphite	*R*_int_ = 0.058
ω scans	θ_max_ = 27.5°, θ_min_ = 3.2°
Absorption correction: multi-scan (*ABSCOR*; Higashi, 1995)	*h* = −13→14
*T*_min_ = 0.981, *T*_max_ = 0.986	*k* = −24→24
35250 measured reflections	*l* = −22→21

### Refinement

**Table:**

Refinement on *F*^2^	Primary atom site location: structure-invariant direct methods
Least-squares matrix: full	Secondary atom site location: difference Fourier map
*R*[*F*^2^ > 2σ(*F*^2^)] = 0.051	Hydrogen site location: inferred from neighbouring sites
*wR*(*F*^2^) = 0.142	H-atom parameters constrained
*S* = 1.01	*w* = 1/[σ^2^(*F*_o_^2^) + (0.0732*P*)^2^ + 0.0076*P*] where *P* = (*F*_o_^2^ + 2*F*_c_^2^)/3
8285 reflections	(Δ/σ)_max_ = 0.001
480 parameters	Δρ_max_ = 0.14 e Å^−^^3^
0 restraints	Δρ_min_ = −0.19 e Å^−^^3^

### Fractional atomic coordinates and isotropic or equivalent isotropic displacement parameters (Å^2^)

**Table:**

	*x*	*y*	*z*	*U*_iso_*/*U*_eq_	
C1	0.67816 (14)	0.26248 (9)	0.59902 (9)	0.0395 (4)	
C2	0.76343 (16)	0.18804 (10)	0.53310 (9)	0.0458 (4)	
C3	0.84043 (18)	0.15308 (11)	0.49281 (11)	0.0593 (5)	
H3	0.9179	0.1704	0.4928	0.071*	
C4	0.7980 (2)	0.09211 (12)	0.45300 (12)	0.0679 (6)	
H4	0.8478	0.0678	0.4257	0.081*	
C5	0.6827 (2)	0.06613 (11)	0.45271 (11)	0.0623 (6)	
H5	0.6572	0.0246	0.4253	0.075*	
C6	0.60416 (18)	0.09983 (10)	0.49185 (11)	0.0540 (5)	
H6	0.5264	0.0826	0.4911	0.065*	
C7	0.64802 (15)	0.16091 (9)	0.53243 (9)	0.0431 (4)	
C8	0.46769 (15)	0.20601 (9)	0.57811 (10)	0.0448 (4)	
C9	0.42339 (19)	0.14782 (11)	0.61197 (13)	0.0623 (5)	
H9	0.4751	0.1114	0.6341	0.075*	
C10	0.3007 (2)	0.14498 (14)	0.61229 (15)	0.0766 (7)	
H10	0.2692	0.1057	0.6337	0.092*	
C11	0.2254 (2)	0.19923 (16)	0.58148 (16)	0.0827 (8)	
H11	0.1433	0.1971	0.5830	0.099*	
C12	0.26996 (19)	0.25670 (14)	0.54838 (14)	0.0765 (7)	
H12	0.2184	0.2939	0.5283	0.092*	
C13	0.39177 (16)	0.25975 (11)	0.54466 (11)	0.0580 (5)	
H13	0.4216	0.2976	0.5199	0.070*	
C14	0.47156 (15)	0.39957 (9)	0.78240 (9)	0.0405 (4)	
C15	0.31701 (16)	0.38001 (11)	0.84005 (10)	0.0493 (4)	
C16	0.22712 (19)	0.35362 (13)	0.87530 (13)	0.0697 (6)	
H16	0.2223	0.3053	0.8870	0.084*	
C17	0.1447 (2)	0.40314 (16)	0.89222 (15)	0.0861 (8)	
H17	0.0829	0.3878	0.9161	0.103*	
C18	0.1520 (2)	0.47526 (16)	0.87438 (14)	0.0808 (7)	
H18	0.0937	0.5067	0.8854	0.097*	
C19	0.24308 (19)	0.50124 (12)	0.84088 (12)	0.0654 (6)	
H19	0.2483	0.5496	0.8298	0.078*	
C20	0.32741 (16)	0.45222 (10)	0.82405 (10)	0.0490 (5)	
C21	0.44315 (15)	0.27268 (9)	0.82388 (10)	0.0440 (4)	
C22	0.55981 (17)	0.25387 (11)	0.85886 (11)	0.0539 (5)	
H22	0.6175	0.2888	0.8765	0.065*	
C23	0.5890 (2)	0.18240 (13)	0.86714 (15)	0.0784 (7)	
H23	0.6675	0.1687	0.8897	0.094*	
C24	0.5018 (2)	0.13114 (13)	0.84194 (18)	0.0949 (9)	
H24	0.5219	0.0829	0.8475	0.114*	
C25	0.3868 (2)	0.15060 (12)	0.80906 (16)	0.0817 (7)	
H25	0.3284	0.1156	0.7930	0.098*	
C26	0.35646 (18)	0.22138 (11)	0.79942 (12)	0.0606 (5)	
H26	0.2778	0.2347	0.7765	0.073*	
C27	0.81789 (15)	0.50360 (9)	0.69485 (10)	0.0417 (4)	
C28	0.93525 (15)	0.59210 (9)	0.73459 (10)	0.0473 (4)	
C29	1.00798 (17)	0.64426 (11)	0.77757 (12)	0.0608 (5)	
H29	1.0202	0.6451	0.8309	0.073*	
C30	1.0609 (2)	0.69445 (12)	0.73781 (14)	0.0711 (6)	
H30	1.1105	0.7293	0.7652	0.085*	
C31	1.04256 (19)	0.69455 (12)	0.65845 (14)	0.0702 (6)	
H31	1.0788	0.7300	0.6340	0.084*	
C32	0.97182 (17)	0.64332 (11)	0.61455 (12)	0.0584 (5)	
H32	0.9596	0.6429	0.5612	0.070*	
C33	0.92006 (15)	0.59245 (9)	0.65505 (10)	0.0464 (4)	
C34	0.79088 (15)	0.52221 (9)	0.55055 (10)	0.0446 (4)	
C35	0.6804 (2)	0.55176 (12)	0.52083 (12)	0.0724 (6)	
H35	0.6375	0.5760	0.5525	0.087*	
C36	0.6330 (2)	0.54512 (14)	0.44293 (14)	0.0873 (8)	
H36	0.5576	0.5649	0.4222	0.105*	
C37	0.6958 (2)	0.51002 (13)	0.39669 (13)	0.0755 (7)	
H37	0.6653	0.5077	0.3441	0.091*	
C38	0.8029 (2)	0.47839 (15)	0.42712 (13)	0.0808 (7)	
H38	0.8443	0.4527	0.3956	0.097*	
C39	0.85137 (18)	0.48410 (13)	0.50520 (11)	0.0637 (6)	
H39	0.9246	0.4620	0.5262	0.076*	
C40	0.66199 (14)	0.32475 (9)	0.64708 (9)	0.0388 (4)	
C41	0.57686 (15)	0.32759 (9)	0.69462 (9)	0.0402 (4)	
H41	0.5252	0.2890	0.6962	0.048*	
C42	0.56899 (14)	0.38813 (9)	0.73976 (9)	0.0392 (4)	
C43	0.64966 (15)	0.44444 (9)	0.73921 (9)	0.0421 (4)	
H43	0.6464	0.4840	0.7705	0.051*	
C44	0.73500 (14)	0.44234 (9)	0.69252 (9)	0.0405 (4)	
C45	0.73934 (15)	0.38286 (9)	0.64583 (9)	0.0420 (4)	
H45	0.7947	0.3819	0.6133	0.050*	
C46	0.0678 (2)	0.15090 (15)	0.15807 (17)	0.0937 (8)	
H46A	0.1492	0.1359	0.1572	0.141*	
H46B	0.0246	0.1122	0.1759	0.141*	
H46C	0.0700	0.1911	0.1919	0.141*	
N1	0.78056 (12)	0.25108 (8)	0.57557 (8)	0.0449 (4)	
N2	0.59401 (12)	0.20906 (7)	0.57505 (8)	0.0413 (3)	
N3	0.41101 (12)	0.34643 (8)	0.81320 (8)	0.0439 (3)	
N4	0.42540 (13)	0.46350 (8)	0.78866 (8)	0.0478 (4)	
N5	0.86992 (13)	0.53606 (8)	0.75867 (8)	0.0470 (4)	
N6	0.84508 (12)	0.53411 (8)	0.63005 (8)	0.0444 (4)	
O	0.00881 (14)	0.17053 (11)	0.08356 (10)	0.0876 (5)	
H0	−0.0508	0.1950	0.0860	0.131*	

### Atomic displacement parameters (Å^2^)

**Table:**

	*U*^11^	*U*^22^	*U*^33^	*U*^12^	*U*^13^	*U*^23^
C1	0.0428 (9)	0.0382 (9)	0.0378 (9)	0.0012 (7)	0.0090 (7)	−0.0003 (7)
C2	0.0497 (10)	0.0482 (11)	0.0393 (9)	0.0079 (8)	0.0083 (8)	−0.0039 (8)
C3	0.0529 (11)	0.0696 (14)	0.0574 (12)	0.0098 (10)	0.0159 (9)	−0.0125 (10)
C4	0.0722 (14)	0.0721 (15)	0.0609 (13)	0.0191 (12)	0.0172 (10)	−0.0184 (11)
C5	0.0797 (14)	0.0491 (12)	0.0553 (12)	0.0111 (10)	0.0070 (10)	−0.0160 (10)
C6	0.0656 (12)	0.0432 (11)	0.0517 (11)	0.0025 (9)	0.0079 (9)	−0.0076 (9)
C7	0.0504 (10)	0.0390 (10)	0.0396 (9)	0.0066 (8)	0.0081 (8)	−0.0035 (7)
C8	0.0450 (9)	0.0417 (10)	0.0480 (10)	−0.0021 (8)	0.0104 (8)	−0.0098 (8)
C9	0.0621 (12)	0.0505 (12)	0.0782 (14)	−0.0035 (10)	0.0235 (11)	−0.0029 (11)
C10	0.0691 (14)	0.0708 (16)	0.0987 (18)	−0.0202 (13)	0.0377 (13)	−0.0130 (14)
C11	0.0488 (12)	0.101 (2)	0.0994 (19)	−0.0136 (13)	0.0188 (12)	−0.0282 (16)
C12	0.0515 (12)	0.0854 (18)	0.0883 (16)	0.0151 (12)	0.0035 (12)	−0.0079 (14)
C13	0.0526 (11)	0.0559 (13)	0.0625 (12)	0.0068 (9)	0.0047 (9)	−0.0037 (10)
C14	0.0487 (9)	0.0375 (10)	0.0355 (8)	0.0019 (7)	0.0090 (7)	0.0006 (7)
C15	0.0490 (10)	0.0567 (12)	0.0448 (10)	0.0118 (9)	0.0162 (8)	0.0046 (9)
C16	0.0631 (12)	0.0756 (16)	0.0783 (15)	0.0110 (11)	0.0332 (11)	0.0179 (12)
C17	0.0706 (15)	0.108 (2)	0.0917 (18)	0.0232 (14)	0.0456 (13)	0.0166 (16)
C18	0.0734 (15)	0.095 (2)	0.0805 (16)	0.0333 (14)	0.0306 (12)	−0.0024 (14)
C19	0.0694 (13)	0.0643 (14)	0.0638 (13)	0.0213 (11)	0.0167 (10)	−0.0074 (11)
C20	0.0521 (10)	0.0548 (12)	0.0401 (9)	0.0109 (9)	0.0092 (8)	−0.0036 (8)
C21	0.0508 (10)	0.0389 (10)	0.0453 (10)	0.0034 (8)	0.0168 (8)	0.0070 (8)
C22	0.0520 (10)	0.0542 (12)	0.0542 (11)	0.0034 (9)	0.0080 (9)	0.0081 (9)
C23	0.0648 (14)	0.0624 (15)	0.1063 (19)	0.0190 (12)	0.0131 (13)	0.0255 (14)
C24	0.0898 (18)	0.0445 (14)	0.153 (3)	0.0073 (13)	0.0306 (18)	0.0264 (15)
C25	0.0733 (15)	0.0480 (14)	0.123 (2)	−0.0111 (11)	0.0184 (14)	0.0130 (13)
C26	0.0525 (11)	0.0520 (12)	0.0763 (14)	−0.0045 (9)	0.0106 (10)	0.0096 (10)
C27	0.0444 (9)	0.0367 (9)	0.0432 (9)	0.0019 (7)	0.0067 (7)	−0.0011 (7)
C28	0.0455 (9)	0.0422 (10)	0.0514 (11)	−0.0022 (8)	0.0027 (8)	−0.0037 (8)
C29	0.0599 (12)	0.0555 (13)	0.0618 (12)	−0.0063 (10)	−0.0002 (10)	−0.0134 (10)
C30	0.0667 (13)	0.0565 (14)	0.0844 (16)	−0.0214 (11)	0.0019 (12)	−0.0125 (12)
C31	0.0676 (13)	0.0555 (14)	0.0853 (16)	−0.0236 (10)	0.0098 (12)	0.0035 (12)
C32	0.0594 (11)	0.0550 (12)	0.0596 (12)	−0.0132 (10)	0.0089 (9)	0.0046 (10)
C33	0.0445 (9)	0.0410 (10)	0.0509 (10)	−0.0046 (8)	0.0033 (8)	−0.0010 (8)
C34	0.0504 (10)	0.0397 (10)	0.0419 (9)	−0.0089 (8)	0.0052 (8)	0.0012 (8)
C35	0.0753 (14)	0.0689 (15)	0.0637 (13)	0.0202 (12)	−0.0081 (11)	−0.0104 (11)
C36	0.0959 (18)	0.0762 (17)	0.0717 (16)	0.0146 (14)	−0.0263 (14)	−0.0019 (13)
C37	0.1030 (18)	0.0703 (16)	0.0457 (11)	−0.0252 (14)	−0.0027 (12)	0.0053 (11)
C38	0.0850 (16)	0.106 (2)	0.0542 (13)	−0.0097 (15)	0.0204 (12)	−0.0130 (13)
C39	0.0561 (11)	0.0835 (16)	0.0517 (11)	−0.0008 (11)	0.0110 (9)	−0.0060 (11)
C40	0.0441 (9)	0.0354 (9)	0.0363 (8)	0.0021 (7)	0.0067 (7)	−0.0008 (7)
C41	0.0470 (9)	0.0348 (9)	0.0387 (9)	−0.0035 (7)	0.0081 (7)	−0.0006 (7)
C42	0.0457 (9)	0.0369 (9)	0.0349 (8)	0.0022 (7)	0.0076 (7)	0.0013 (7)
C43	0.0517 (10)	0.0348 (9)	0.0390 (9)	0.0007 (8)	0.0071 (8)	−0.0014 (7)
C44	0.0445 (9)	0.0359 (9)	0.0402 (9)	0.0003 (7)	0.0065 (7)	0.0018 (7)
C45	0.0453 (9)	0.0398 (10)	0.0420 (9)	0.0020 (8)	0.0116 (7)	0.0005 (8)
C46	0.0745 (16)	0.098 (2)	0.105 (2)	0.0080 (14)	0.0076 (15)	−0.0065 (16)
N1	0.0444 (8)	0.0489 (9)	0.0424 (8)	0.0005 (7)	0.0111 (6)	−0.0057 (7)
N2	0.0437 (7)	0.0356 (8)	0.0454 (8)	0.0015 (6)	0.0110 (6)	−0.0041 (6)
N3	0.0468 (8)	0.0423 (9)	0.0453 (8)	0.0048 (7)	0.0154 (6)	0.0039 (7)
N4	0.0598 (9)	0.0402 (9)	0.0437 (8)	0.0059 (7)	0.0107 (7)	−0.0026 (7)
N5	0.0520 (8)	0.0434 (9)	0.0436 (8)	−0.0005 (7)	0.0045 (7)	−0.0029 (7)
N6	0.0494 (8)	0.0406 (8)	0.0414 (8)	−0.0096 (6)	0.0052 (6)	0.0006 (6)
O	0.0620 (10)	0.1121 (15)	0.0919 (12)	0.0045 (9)	0.0231 (9)	−0.0123 (10)

### Geometric parameters (Å, °)

**Table:**

C1—N1	1.319 (2)	C23—H23	0.9300
C1—N2	1.383 (2)	C24—C25	1.357 (3)
C1—C40	1.476 (2)	C24—H24	0.9300
C2—N1	1.392 (2)	C25—C26	1.369 (3)
C2—C7	1.392 (2)	C25—H25	0.9300
C2—C3	1.393 (3)	C26—H26	0.9300
C3—C4	1.375 (3)	C27—N5	1.315 (2)
C3—H3	0.9300	C27—N6	1.373 (2)
C4—C5	1.385 (3)	C27—C44	1.472 (2)
C4—H4	0.9300	C28—C33	1.389 (3)
C5—C6	1.381 (3)	C28—N5	1.395 (2)
C5—H5	0.9300	C28—C29	1.397 (2)
C6—C7	1.387 (2)	C29—C30	1.379 (3)
C6—H6	0.9300	C29—H29	0.9300
C7—N2	1.392 (2)	C30—C31	1.383 (3)
C8—C13	1.374 (3)	C30—H30	0.9300
C8—C9	1.383 (3)	C31—C32	1.382 (3)
C8—N2	1.434 (2)	C31—H31	0.9300
C9—C10	1.383 (3)	C32—C33	1.389 (3)
C9—H9	0.9300	C32—H32	0.9300
C10—C11	1.364 (4)	C33—N6	1.396 (2)
C10—H10	0.9300	C34—C39	1.356 (3)
C11—C12	1.367 (3)	C34—C35	1.366 (3)
C11—H11	0.9300	C34—N6	1.439 (2)
C12—C13	1.387 (3)	C35—C36	1.385 (3)
C12—H12	0.9300	C35—H35	0.9300
C13—H13	0.9300	C36—C37	1.356 (4)
C14—N4	1.316 (2)	C36—H36	0.9300
C14—N3	1.378 (2)	C37—C38	1.354 (3)
C14—C42	1.468 (2)	C37—H37	0.9300
C15—C16	1.383 (3)	C38—C39	1.389 (3)
C15—C20	1.389 (3)	C38—H38	0.9300
C15—N3	1.394 (2)	C39—H39	0.9300
C16—C17	1.385 (3)	C40—C45	1.395 (2)
C16—H16	0.9300	C40—C41	1.400 (2)
C17—C18	1.391 (4)	C41—C42	1.400 (2)
C17—H17	0.9300	C41—H41	0.9300
C18—C19	1.373 (3)	C42—C43	1.391 (2)
C18—H18	0.9300	C43—C44	1.390 (2)
C19—C20	1.394 (3)	C43—H43	0.9300
C19—H19	0.9300	C44—C45	1.394 (2)
C20—N4	1.391 (2)	C45—H45	0.9300
C21—C26	1.375 (3)	C46—O	1.405 (3)
C21—C22	1.380 (2)	C46—H46A	0.9600
C21—N3	1.428 (2)	C46—H46B	0.9600
C22—C23	1.377 (3)	C46—H46C	0.9600
C22—H22	0.9300	O—H0	0.8200
C23—C24	1.381 (3)		
			
N1—C1—N2	111.94 (14)	C21—C26—H26	120.3
N1—C1—C40	121.80 (15)	N5—C27—N6	113.30 (15)
N2—C1—C40	126.23 (15)	N5—C27—C44	123.57 (16)
N1—C2—C7	109.91 (15)	N6—C27—C44	123.10 (14)
N1—C2—C3	130.03 (17)	C33—C28—N5	110.53 (15)
C7—C2—C3	120.03 (17)	C33—C28—C29	119.45 (18)
C4—C3—C2	117.74 (19)	N5—C28—C29	130.02 (17)
C4—C3—H3	121.1	C30—C29—C28	117.4 (2)
C2—C3—H3	121.1	C30—C29—H29	121.3
C3—C4—C5	121.40 (19)	C28—C29—H29	121.3
C3—C4—H4	119.3	C29—C30—C31	122.09 (19)
C5—C4—H4	119.3	C29—C30—H30	119.0
C6—C5—C4	122.13 (19)	C31—C30—H30	119.0
C6—C5—H5	118.9	C32—C31—C30	121.7 (2)
C4—C5—H5	118.9	C32—C31—H31	119.2
C5—C6—C7	116.12 (18)	C30—C31—H31	119.2
C5—C6—H6	121.9	C31—C32—C33	115.9 (2)
C7—C6—H6	121.9	C31—C32—H32	122.1
C6—C7—N2	131.78 (17)	C33—C32—H32	122.1
C6—C7—C2	122.57 (17)	C28—C33—C32	123.47 (17)
N2—C7—C2	105.56 (14)	C28—C33—N6	105.26 (15)
C13—C8—C9	121.05 (18)	C32—C33—N6	131.27 (17)
C13—C8—N2	119.30 (17)	C39—C34—C35	120.61 (17)
C9—C8—N2	119.61 (16)	C39—C34—N6	119.98 (16)
C10—C9—C8	118.6 (2)	C35—C34—N6	119.37 (17)
C10—C9—H9	120.7	C34—C35—C36	119.2 (2)
C8—C9—H9	120.7	C34—C35—H35	120.4
C11—C10—C9	120.7 (2)	C36—C35—H35	120.4
C11—C10—H10	119.7	C37—C36—C35	120.4 (2)
C9—C10—H10	119.7	C37—C36—H36	119.8
C10—C11—C12	120.3 (2)	C35—C36—H36	119.8
C10—C11—H11	119.9	C38—C37—C36	120.0 (2)
C12—C11—H11	119.9	C38—C37—H37	120.0
C11—C12—C13	120.3 (2)	C36—C37—H37	120.0
C11—C12—H12	119.9	C37—C38—C39	120.3 (2)
C13—C12—H12	119.9	C37—C38—H38	119.8
C8—C13—C12	119.0 (2)	C39—C38—H38	119.8
C8—C13—H13	120.5	C34—C39—C38	119.4 (2)
C12—C13—H13	120.5	C34—C39—H39	120.3
N4—C14—N3	112.69 (15)	C38—C39—H39	120.3
N4—C14—C42	121.61 (15)	C45—C40—C41	119.02 (15)
N3—C14—C42	125.42 (15)	C45—C40—C1	117.07 (15)
C16—C15—C20	122.57 (18)	C41—C40—C1	123.89 (15)
C16—C15—N3	131.95 (19)	C40—C41—C42	120.39 (15)
C20—C15—N3	105.47 (16)	C40—C41—H41	119.8
C15—C16—C17	116.3 (2)	C42—C41—H41	119.8
C15—C16—H16	121.9	C43—C42—C41	119.42 (16)
C17—C16—H16	121.9	C43—C42—C14	116.70 (15)
C16—C17—C18	121.7 (2)	C41—C42—C14	123.57 (15)
C16—C17—H17	119.2	C44—C43—C42	120.85 (16)
C18—C17—H17	119.2	C44—C43—H43	119.6
C19—C18—C17	121.7 (2)	C42—C43—H43	119.6
C19—C18—H18	119.2	C43—C44—C45	119.25 (15)
C17—C18—H18	119.2	C43—C44—C27	118.10 (15)
C18—C19—C20	117.4 (2)	C45—C44—C27	122.66 (16)
C18—C19—H19	121.3	C44—C45—C40	121.01 (16)
C20—C19—H19	121.3	C44—C45—H45	119.5
C15—C20—N4	110.29 (15)	C40—C45—H45	119.5
C15—C20—C19	120.39 (19)	O—C46—H46A	109.5
N4—C20—C19	129.27 (19)	O—C46—H46B	109.5
C26—C21—C22	120.99 (17)	H46A—C46—H46B	109.5
C26—C21—N3	119.12 (16)	O—C46—H46C	109.5
C22—C21—N3	119.88 (16)	H46A—C46—H46C	109.5
C23—C22—C21	118.72 (19)	H46B—C46—H46C	109.5
C23—C22—H22	120.6	C1—N1—C2	105.86 (14)
C21—C22—H22	120.6	C1—N2—C7	106.72 (14)
C22—C23—C24	120.0 (2)	C1—N2—C8	129.74 (14)
C22—C23—H23	120.0	C7—N2—C8	122.62 (13)
C24—C23—H23	120.0	C14—N3—C15	106.30 (14)
C25—C24—C23	120.5 (2)	C14—N3—C21	128.04 (15)
C25—C24—H24	119.7	C15—N3—C21	125.35 (15)
C23—C24—H24	119.7	C14—N4—C20	105.23 (15)
C24—C25—C26	120.3 (2)	C27—N5—C28	104.64 (14)
C24—C25—H25	119.8	C27—N6—C33	106.26 (14)
C26—C25—H25	119.8	C27—N6—C34	129.54 (14)
C25—C26—C21	119.42 (19)	C33—N6—C34	122.91 (14)
C25—C26—H26	120.3	C46—O—H0	109.5

### Hydrogen-bond geometry (Å, °)

**Table:**

*D*—H···*A*	*D*—H	H···*A*	*D*···*A*	*D*—H···*A*
O—H0···N1^i^	0.82	2.12	2.936 (2)	170
C25—H25···N5^ii^	0.93	2.69	3.603 (3)	169
C37—H37···N4^iii^	0.93	2.43	3.338 (3)	164
C32—H32···O^iv^	0.93	2.71	3.605 (3)	161

Symmetry codes: (i) *x*−1, −*y*+1/2, *z*−1/2; (ii) −*x*+1, *y*−1/2, −*z*+3/2; (iii) −*x*+1, −*y*+1, −*z*+1; (iv) −*x*+1, *y*+1/2, −*z*+1/2.

## References

[bb1] Adachi, C., Baldo, M. A., Forrest, S. R., Lamansky, S., Thompson, M. E. & Kwong, R. C. (2001). *Appl. Phys. Lett.* **78**, 1622–1624.

[bb2] Gao, Z., Lee, C. S., Bello, I., Lee, S. T., Chen, R.-M., Luh, T.-Y., Shi, J. & Tang, C. W. (1999). *Appl. Phys. Lett.* **74**, 865–867.

[bb3] Higashi, T. (1995). *ABSCOR* Rigaku Corporation, Tokyo, Japan.

[bb4] Lo, S.-C., Male, N. A. H., Markham, J. P. J., Magennis, S. W., Burn, P. L., Salata, O. V. & Samuel, I. D. W. (2002). *Adv. Mater.* **14**, 975–979.

[bb5] Rigaku (1998). *RAPID-AUTO* Rigaku Corporation, Tokyo, Japan.

[bb6] Sheldrick, G. M. (2008). *Acta Cryst.* A**64**, 112–122. 10.1107/S0108767307043930 18156677

[bb7] Shi, J., Tang, C. W. & Chen, C. H. (1997). US Patent 5645948.

[bb8] Totsatitpaisan, P., Tashiro, K. & Chirachanchai, S. (2008). *J. Phys. Chem. A*, **112**, 10348–10358. 10.1021/jp8045774 18816029

